# A Prolonged Norovirus Infection and the Molecular Evolution of Human Norovirus Within-Host in a Child with Burkitt Lymphoma

**DOI:** 10.3390/v18050538

**Published:** 2026-05-07

**Authors:** Liping Jia, Ri De, Zeng Li, Zhenzhi Han, Liying Liu, Huijin Dong, Shunqiao Feng, Rong Liu, Linqing Zhao

**Affiliations:** 1 Laboratory of Virology, Capital Health Center for Children’s Health, Capital Institute of Pediatrics, Capital Medical University, Beijing 100020, China; im_jiaping@126.com (L.J.); graceride@163.com (R.D.); drlzeng@163.com (Z.L.); liuliying1201@163.com (L.L.); xiaoabo03@163.com (H.D.); 2 Department of Hematology, Capital Health Center for Children’s Health, Capital Medical University, Beijing 100020, China; fengshunqiao@163.com

**Keywords:** human norovirus, molecular evolution, Burkitt lymphoma, intra-host quasi-species, GII.4 Sydney_2012

## Abstract

It has been reported that chronic infection of human norovirus (HuNoV) may potentially serve as a reservoir for viral variants with the possibility to evade population immunity or alter the binding sites of HBGA receptors. In this study, a child diagnosed with Burkitt lymphoma and positive for HuNoV determined by real-time PCR (qPCR) firstly in 15 August 2016, was followed up until 20 March 2018, and 26 fecal specimens and one vomitus were collected to trace the evolutionary characteristics of HuNoV by phylogenetic analysis, meta-genomics next-generation sequencing (mNGS), and temporal evolutionary analysis of VP1 among 23 specimens positive for HuNoV. There were 15 specimens with partial RdRp gene sequences forming an independent cluster with sequences of GII.P31, 14 with the region C sequences and 11 with P domain sequences of VP1 gene clustered together with HuNoV GII.4 Sydney_2012. All these sequences showed that mutations accumulated nearly in a time order, and more mutations were shown in the key epitopes A–E or near the binding sites for HBGA in subdomain P2 with higher evolutionary rates. Analysis of NGS data identified intra-host viral quasi-species, and two genome sequences of the same length from mNGS were assembled from N705, with mutations located in the region of subdomain P2 (1171 nt–1202 nt) which led to five amino acid mutations. In conclusion, the accumulated mutations of HuNoV, especially in subdomain P2, were explored in a child with Burkitt lymphoma, and the sequencing of HuNoV from immunocompromised individuals was proven critical for monitoring intra-host quasi-species evolution and potential variant emergence, providing basic data for clinical infection control.

## 1. Introduction

Human norovirus (HuNoV) belongs to the genera Norovirus in the family Caliciviridae, with a single strand, positive-sense RNA genome of approximately 7.5~7.7 kb in length, which contains three open reading frames (ORF1–3) [1]. ORF1 encodes a non-structural polyprotein that is post-translationally cleaved by the viral protease into six non-structural proteins, and the viral RNA-dependent RNA polymerase (RdRp) is one of them. ORF2 and ORF3 are translated from a subgenomic RNA that encodes the major (VP1) and minor (VP2) capsid proteins, respectively. The VP1 consists of a capsid (S) and a protruding (P) domain, connected by a hinge which is composed of eight amino acids (aa) [2]. Moreover, the P domain is divided into subdomains P1 and P2. The key antigenic sites (A, C, D, E, F, G, and I) that play a major role in the immunodominance of hosts’ responses to GII.4 noroviruses are mapped to the surface-exposed P2 subdomain and they also interact strongly with cellular histo-blood group antigens (HBGA) [3,4,5,6,7,8]. One of the major obstacles for HuNoV vaccine development is the large viral genetic and antigenic diversity. HuNoV genomic replication is accomplished by the viral RdRP. Although norovirus RdRPs have low fidelity, mutation accumulation varies across genotypes, and no significant differences in substitution rates have been demonstrated across genotypes or genomic regions [9,10]. Genetic differences within VP1 in the amino acid level have been used to classify the virus into genogroups GI-GX and more than 50 genotypes, in which only GI, GII, GIV and GIX can infect human beings. Among these genotypes, GII.4 has been the dominant genotype worldwide for nearly 30 years. However, other genotypes (e.g., GII.2 and GII.17) have also been predominant in specific regions and time periods [11,12,13,14]. Notably, the rapid turnover of GII.4 variants (2–4 years) was only observed during 2002–2012; US 95/96 and Sydney 2012 variants have circulated for more than 7 years without variant replacement [12]. The diversity of the RdRp region of ORF1 in the nucleotide level has been used to obtain 60 P-types [15]. Therefore, a dual typing model based on VP1 amino acid and RdRp nucleotide diversity is commonly used now.

HuNoVs are the leading cause of acute gastroenteritis (AGE) and are estimated to cause 677 million cases of AGE globally each year [16]. Though all populations are susceptible to the virus, the greatest disease burden is in children under 5 years of age. Symptoms of AGE caused by HuNoV include vomiting, diarrhea, cramps, and abdominal pain, which typically manifest 12 to 48 h following the contact with contaminated food or infected persons and are usually mild and self-limiting [16]. However, the elderly, the very young and immunocompromised patients are under increased risk for severe and life-threatening illness, in which HuNoV often leads to chronic and persistent infections lasting for several weeks to several years [17,18,19,20]. In chronic and persistent infections, mutations often accumulate in the viral genome due to the prolonged replication cycle. Therefore, patients with chronic HuNoV infection may potentially be a reservoir for viral variants that could evade population immunity or alter the binding sites of HBGA receptors [20]. In this study, a child diagnosed with Burkitt lymphoma and positive for HuNoV, as determined by real-time PCR (qPCR) firstly on 15 August 2016, was followed up until 20 March 2018, and 26 fecal specimens and one vomitus were collected to trace the evolutionary characteristics of HuNoV genome in chronic infection, combined with NGS analysis to characterize intra-host viral quasi-species diversity.

## 2. Materials and Methods

### 2.1. The Case Enrolled

On 30 March 2016, a 2-year-old boy was admitted to Capital Health Center for Children’s Health, Capital Medical University, for the first time because of an abdominal mass with intermittent abdominal pain, and then diagnosed with Burkitt lymphoma by pathological biopsy. His ileum tumor was removed, and chemotherapy was chosen on 8 June 2016. In November 2016, the boy underwent the autologous hematopoietic stem cell transplantation.

During the hospitalization, the respiratory pathogens screened from respiratory specimens and Epstein–Barr virus (EBV) and cytomegalovirus (CMV) in plasma were all negative. With the occurrence of watery stools since 18 July 2016, and vomiting since 25 July 2016, white blood cells appeared in routine stool tests. Intermittent vomiting with watery stools continued until 20 December 2016; during this period, there was occasional fever but no abdominal pain.

On 15 August 2016, the first fecal specimen was sent to our laboratory for the nucleic acid detection of HuNoV, which showed a positive result. Then, the nucleic acid of HuNoV was continuously monitored to observe its shedding time and evolution in the host with chronic HuNoV infection, until three consecutive specimens showed negative results.

### 2.2. Nucleic Acid Detection

The stool and vomitus specimens collected from the boy were prepared as 10% (*w*/*v*) suspensions in phosphate-buffered saline (PBS) and then centrifuged for 5 min at 5000× *g*. Then, total nucleic acid was extracted from 140 µL supernatant of each specimen using the QIAamp Viral RNA Mini Kit (Qiagen, Hilden, Germany) according to the manufacturer’s instructions. The RNA of HuNoV was detected using a real-time reverse-transcription polymerase chain reaction (rRT-PCR) Kit (Land medical, Wuhan, China) on System LC480 (Roche, Basel, Switzerland). According to the instruction manual, specimens with cycle threshold values (Ct) < 36 were considered positive for HuNoV, while specimens with cycle Ct ≥ 36 were considered negative for HuNoV.

### 2.3. Reverse Transcription PCR for HuNoV Genotyping and P Domain Sequencing

The extracted nucleic acids were used as templates to synthesize cDNA by the conventional two-step reverse transcription reaction (RT) with random primers. Moloney Murine Leukemia virus (M-MLV) reverse transcriptase (200 U/μL) (Invitrogen, Carlsbad, CA, USA), Ribonuclease Inhibitor (TransGen Biotech, Beijing, China), and dNTP (TransGen Biotech, Beijing, China) were added separately to reaction mixtures according to the manufacturer’s instructions.

The primer sets GIISKF/GIISKR and P289/P290 were used for PCR to amplify region C of VP1 and RdRp region for G genotyping and P typing, respectively, by sequencing using the online Typing Tool (http://www.rivm.nl/mpf/norovirus/typingtool, accessed on 1 April 2019) [21,22,23].

Sanger sequencing was used for partial gene fragment amplification in this study; the domain P of VP1 was amplified using the primer sets P422/P409 by PCR [24], which was then purified and sequenced by the Sino GenoMax Co., Ltd. (Beijing, China).

### 2.4. Phylogenetic and Evolutionary Analysis

Lasergene’s DNA SeqMan software (version 7.1.0, DNA Star Inc., Madison, WI, USA) was used to assemble nucleotide sequences of RdRp gene and domain P of VP1. All sequences were named as follows: No.-date of specimen collection. The MAFFT software version 7.49 was adopted to align sequences. Phylogenetic analyses were carried using the MEGA 6.0 program, in which the sequence identity and evolutionary divergence of these sequences were estimated, and the phylogenetic trees of each gene were constructed using the maximum likelihood (ML) method with 1000 bootstrap replicates [25]. Reference sequences were downloaded from the National Center for Biotechnology Information (NCBI). Evolutionary rates were inferred using Bayesian MCMC in BEAST v1.10.4. The best-fitting model was selected with Model Finder, and optimal clock/tree prior combinations were chosen via Bayes Factor comparison [26].

### 2.5. Amino Acid Mutation Analysis of Subdomain P2 of HuNoV VP1

Moreover, to understand the evolutionary characteristics of the highly variable subdomain P2, the key antigenic sites (A, B, C, D, E, F, G, and I) of HuNoV and the binding sites to HBGA, the nucleotide sequences of the subdomain P2 obtained in this study were converted into amino acids using DNAstar software version 7.1.0 and then compared with reference sequences downloaded from NCBI using MEGA 6.0 software.

### 2.6. Meta-Genomics Next-Generation Sequencing (mNGS)

There were three specimens selected for mNGS to confirm the results of phylogenetic analyses. The first one was collected on August 15, the other one had consistence mutations in the amino acid level of the P2 subdomain, and another one was collected in between the collection dates of the other two specimens. For mNGS, viral RNA was extracted from 200 µL of each fecal specimen and eluted in 90 µL elution buffer by the KingFisher Flex Purification System (Thermo Fisher, Waltham, MA, USA), which was followed by cDNA synthesis using random hexamers and the LunaScript RT SuperMix Kit (New England Biolabs, Abingdon, UK) according to the manufacturer’s instructions. Subsequently, primer schemes and Q5 High-Fidelity DNA polymerase (New England Biolabs, Abingdon, UK) were used for HuNoV whole-genome multiplex-PCR amplification. The PCR products were used to prepare libraries for sequencing using a Nextera XT DNA Sample Preparation and Index kit and DNA Prep Sample Preparation and Index kit (Illumina, San Diego, CA, USA) following the manufacturer’s instructions, and the sequencing was carried out on an Illumina MiSeq or MiniSeq platform using the 2 × 150 cycles paired-end sequencing protocol [27].

## 3. Results

### 3.1. Nucleic Acid Detection in the Monitoring Surveillance

In the study, twenty-six fecal and one vomitus specimens were collected from the boy with Burkitt lymphoma between 15 August 2016, and 20 March 2018, for nucleic acid detection by rRT-PCR, in which there were 22 fecal and one vomitus specimens positive for HuNoV RNA with the Ct values increasing slowly from 15.9 to 35 in the fecal specimens. A higher Ct value was shown in the vomitus compared to that in the fecal specimens (Table 1).

Among these 23 specimens, there were 15 with partial RdRp sequences, 14 with region C and 11 with domain P sequences of VP1. All three gene fragments were amplified successfully in nine of ten specimens with Ct values lower than 25 in rRT-PCR. By the online analyses of the region C of VP1 and partial RdRp sequences, all these sequences were identified as GII.4-sydney [P31].

### 3.2. Phylogenetic Analysis of the Domain P of VP1 and Partial RdRp

**Figure 1:**
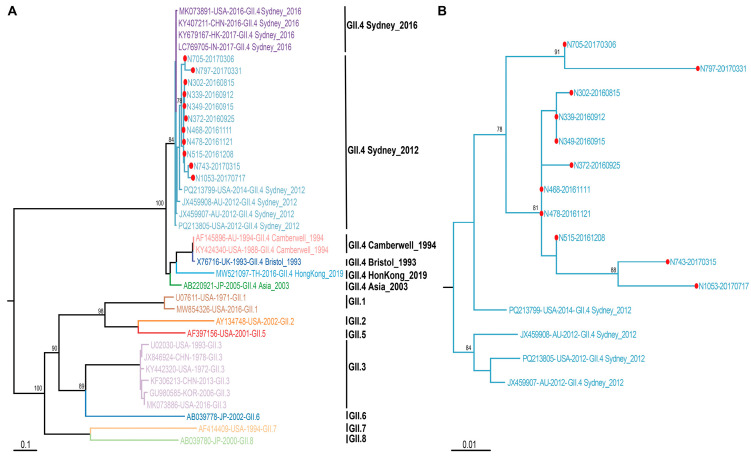
The phylogenetic tree constructed on the basis of the amino acid sequence of domain P of HuNoV VP1. (**A**): All sequences in the phylogenetic tree. (**B**): Only sequences of GII.4 Sydney_2012. The sequences from specimens collected in the study are marked by red solid circles and labeled with N0.-date of collection.

To reveal the molecular evolution of HuNoV in the same host, the domain P nucleotide sequences of VP1 successfully obtained from 11 specimens positive for HuNoV were translated into amino acids and a phylogenetic tree was constructed by comparing these sequences with reference sequences downloaded from NCBI (Figure 1) using MEGA6.0 software. These 11 sequences shared 91–100% sequence identity with the sequence from the specimen N302 that was first collected on 15 August 2016, with more mutations shown in subdomain P2, sharing 88.9–100% of the sequence identity. In the phylogenetic tree of domain P, these 11 sequences were grouped into the cluster of HuNoV GII.4 Sydney_2012 (Figure 1), which was shown nearly in a time order, except those of N705-20170306 and N797-20170331.

**Figure 2:**
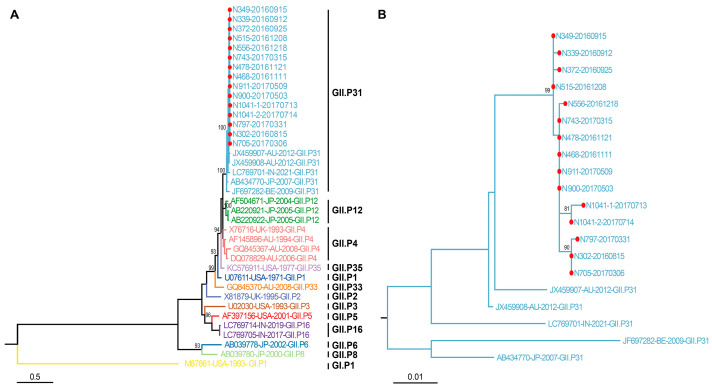
The phylogenetic tree was constructed on the basis of the 15 partial nucleotide sequences of the RdRp gene collected in this study, indicated by red solid circles, which were compared with sequences downloaded from NCBI. (**A**): All sequences in the phylogenetic tree. (**B**): Only sequences of GII.P31.

Then, another phylogenetic tree was constructed using the 15 partial RdRp nucleotide sequences in this study and the reference sequences downloaded from NCBI using MEGA6.0 software. The results indicated that these 15 sequences shared high levels of sequence identity (99.2–100%) with each other and formed an independent cluster in the sequences of GII.P31, which are also shown nearly in a time order (Figure 2).

Using the best-fit nucleotide substitution model JC and an uncorrelated relaxed molecular clock model in BEAST, the evolutionary rate of RdRp gene was estimated to be 1.25 × 10^−3^ (95% HPD: 2.38 × 10^−8^–3.64 × 10^−3^) substitutions/site/year, while that of domain P was calculated as 1.25 × 10^−2^ (95% HPD: 3.80 × 10^−3^–2.13 × 10^−2^) substitutions/site/year.

### 3.3. Amino Acid Sequence Analysis of the Hypervariable Subdomain P2

**Figure 3:**
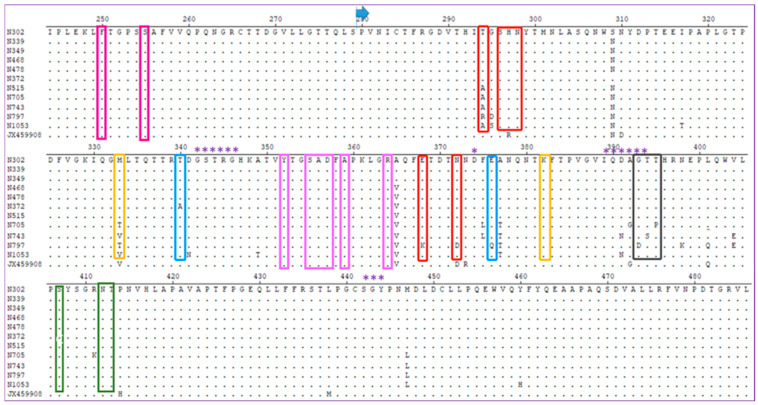
Amino acid sequence alignment of the subdomain P2 of HuNoV VP1 gene collected in the study. Epitope A (red), B (yellow), C (blue), D (black), and E (green), G (pink), I (purple). Numbers on the top indicate the amino acid locations of VP1. * means HBGA binding site. Blue horizontal thick arrows represent the starting of subdomain P2.

The deduced amino acids of the hypervariable subdomain P2 from 11 specimens were aligned with the reference sequences (Figure 3). The alignment revealed a complex mutation pattern rather than simple directional accumulation. Some mutations persisted throughout infection, while others appeared only transiently and reverted to the ancestral sequence. Persistent mutations (positions 294 Thr → Ala/Arg, 365 Ala → Val, and 377 Ala → Thr) appeared early and remained throughout infection. Transient mutations (340 Thr → Ala in N372 only, 368 Ser → Asn in N478/N515, and 372 Ile → Val in N372/N468) appeared only in intermediate samples and reverted. Late-appearing persistent mutations (309 Ser → Asn, 317 Ile → Thr, 341 Asp → Asn, 349 Ala → Thr, 394 Thr → Ser, 447 Met → Ile, and 460 Tyr → His) emerged after month 7 and persisted. More stable mutations were located in epitopes A-E or near HBGA binding sites: position 294 (epitope A; Thr → Ala/Arg), 333 (epitope B; Met → Thr/Val), 340 (epitope C; Thr → Ala, transient), 394 (epitope D; Thr → Ser), and 411 (epitope E; Arg → Lys); a mutation at position 377 (Ala → Thr) occurred near the HBGA binding site. Epitopes G and I were highly conserved across all specimens. The analysis of physical and chemical properties showed that Thr had the highest mutation frequency, followed by Ala (Table 2).

### 3.4. Genomic Sequence Analysis of HuNoV

**Figure 4:**
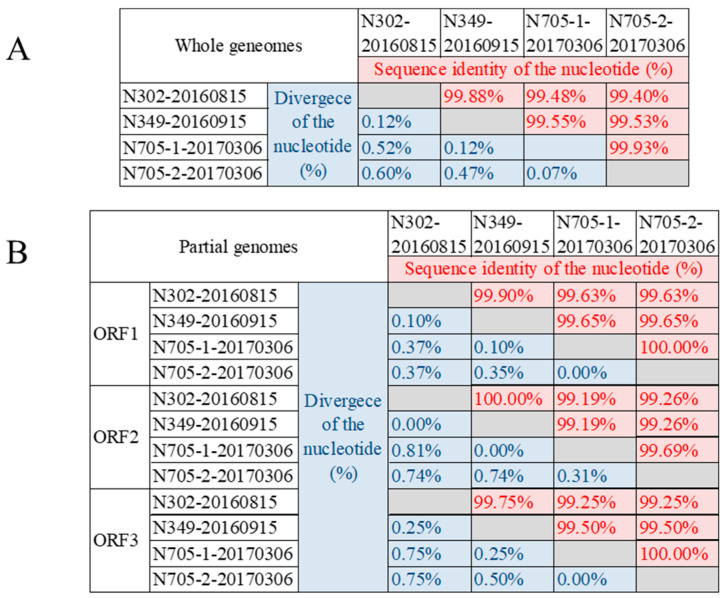
Sequence identity analyses of four genomic sequences from three specimens subjected to mNGS. (**A**) Sequence identity analysis of the whole genomic sequences. (**B**) Sequence identity analysis of ORFs.

Three samples N302, N349 and N705, positive for HuNoV, were subjected to mNGS. Notably, Sanger sequencing chromatograms of the P domain from sample N705 revealed double peaks at several nucleotide positions within the P2 subdomain (Supplementary Figure S1), indicating the presence of mixed viral populations. Consistent with this observation, two distinct genome sequences of the same length were unexpectedly assembled from this specimen, designated N705-1-20170306 and N705-2-20170306. These four HuNoV genomic sequences were 7509 nucleotides (nt) in length, including ORF1 (1–5100 nt), ORF2 (5081–6703 nt) and ORF3 (6703–7509 nt) with a 21 nt overlap between ORF1 and ORF2 and a single nucleotide overlap between ORF2 and ORF3. These four genomic sequences have been submitted to GenBank with accession numbers PP498986–PP498989, which shared high sequence identities with each other (99.47–99.93%) (Figure 4A). Higher sequence identities were shown in genomic sequences between the N302-20160815 and N349-20160915 (99.88%), as well as that between N705-1-20170306 and N705-2-20170306 (99.93%). The genomic sequences of N705-1-20170306 shared higher identity with genomic sequence N302-20160815 and N349-202160915 (99.48% and 99.53%, respectively) than that of N705-2-20170306 (99.47% and 99.53%, respectively). Given that different ORFs of HuNoV have different functions and characteristics, sequence identity analyses of different ORFs were separately conducted (Figure 4B). The ORF1 coding region shared 99.63–100%, the ORF2 coding region shared 99.19–100%, and the ORF3 coding region shared 99.25–100% identity among these four genomic sequences. However, more mutations were shown in the ORF2 coding region with a maximum difference of 0.81%, which was located in the region from 880 nt to 1339 nt, just in domain P. In particular, mutations between N705-1-20170306 and N705-2-20170306 were shown only in the ORF2 coding region with a difference of 0.31%, and were located in the region of subdomain P2 (1171 nt–1202 nt) and led to five amino acid mutations.

## 4. Discussion

In this study, we characterized the intra-host evolution of HuNoV GII.4 Sydney_2012 [P31] in a pediatric Burkitt lymphoma patient over 19 months. The child was infected with HuNoV following ileal tumor resection, and viral shedding persisted for 19 months, with Ct values fluctuating before ultimately exceeding the detection threshold. This pattern suggests a dynamic interplay between viral replication and the host‘s weakened immune responses.

It has been reported that chronic HuNoV infection in immunocompromised patients provides a unique environment for nucleotide and amino acid mutation accumulation, which may potentially lead to new viral variants [28]. The P2 subdomain of VP1 is exposed on the viral capsid surface as a binding site for HBGA and can be involved in escaping neutralizing antibodies [4,29]. In this study, sequences were identified as GII.4 Sydney_2012 [P31], with evolutionary rates of approximately 1.25 × 10^−3^ and 1.25 × 10^−2^ substitutions/site/year in the RdRp region and P domain, respectively. These rates are consistent with the global population evolutionary rate of HuNoV GII.4 [7,10], indicating that the increased mutations in chronic infection are due to the prolonged replication cycle rather than an increased intrinsic mutation rate. More mutations were observed in the P2 subdomain, which shared 88.9–100% sequence identity among specimens, a common characteristic of chronic HuNoV infection in immunocompromised individuals [30,31].

Previous studies have reported varying evolutionary rates for GII.4 VP1: 4.95 × 10^−4^~5.91 × 10^−4^ substitutions/site/year from 1966 to 2019 [32], 7.68 × 10^−3^ (95% HPD; 6.69~8.59 × 10^−3^) from 1974 to 2015 [33], and 4.74~4.95 × 10^−4^ from 2004 to 2015 in China [34]. In the VP1 gene, the average evolutionary rate of the P2 subdomain (9.15 × 10^−3^) was higher than that of domain S (6.97 × 10^−3^) and subdomain P1 (5.79 × 10^−3^). The evolutionary rates observed in our study were higher than previously reported, which may be due to the random drift of viral clonal populations within a single host over an extended period.

However, the hypothesis that chronically infected patients serve as reservoirs for emerging norovirus variants has been challenged. As noted [35], the probability that rare intra-host variants successfully transmit and establish new epidemic lineages remains low, and direct evidence supporting such reservoir-driven emergence is currently lacking. Nonetheless, the continued surveillance of immunocompromised patients with a chronic norovirus infection is warranted to monitor intra-host viral evolution and to better understand the conditions under which variant emergence might occur.

Then four genomic sequences were harvested by mNGS from three specimens: N302, N349 and N705. The evolutionary analysis revealed that the highest variable region was the subdomain P2 of ORF2, which was consistent with the results of VP1 analysis. In particular, two distinct genomic sequences, N705-1-20170306 and N705-2-20170306, harvested from one sample, N705, suggested the intra-host viral quasi-species evolution. N705-1-20170306 showed higher identity with the earlier sequences N302-20160815 (99.48%) and N349-20160915 (99.53%) than N705-2-20170306 did (99.47% with N302-20160815 and 99.53% with N349-20160915), with the highest mutation frequency (up to 0.81% divergence) shown in the ORF2. All five amino acid mutations which can distinguish N705-1-20170306 and N705-2-20170306 were confined to the subdomain P2 in ORF2. This observation is further corroborated by the double peaks observed in the Sanger chromatograms of the P2 domain from the same specimen (Supplementary Figure S1). Together, these multiple lines of evidence confirm the coexistence of distinct intra-host clonal populations during chronic infection. Therefore, the accumulated mutations in subdomain P2 should be monitored more cautiously, especially in immunocompromised patients, the reservoir of new variants of HuNoV [36].

The sequence alignment of the P2 hypervariable subdomain from 11 HuNoV strains revealed a complex mutation pattern rather than simple directional accumulation. Some mutations persisted throughout infection (e.g., positions 294, 365, and 377), while others appeared transiently and reverted to the ancestral sequence (e.g., positions 340, 368, and 372). Additional mutations emerged after December 2016 and persisted thereafter. This pattern is consistent with intra-host viral quasi-species dynamics, where multiple variants compete under fluctuating selective pressures, including immune responses and receptor availability [30]. Most fixed substitutions were located in epitopes A–E and near the HBGA binding site, supporting the hypothesis that antigenic drift facilitates escape from host immune pressure [37]. In contrast, epitopes G and I were highly conserved; this observation aligns with [12], who found that epitope G remained conserved after a decade of global circulation of GII.4 Sydney_2012. Unlike epitopes A–E, which tolerate immune-escape mutations, demonstrating that epitope G may be under strong structural constraints critical for viral fitness. This highlights the potential of targeting conserved epitopes for vaccine development [38]. Clinically, the patient’s AGE symptoms occurred mainly within the first five months, and the child was thereafter asymptomatic despite continued viral shedding. No temporal correlation was found between specific P2 mutations and symptom recurrence. Thus, while these mutations may alter antigenicity or receptor binding, they were clinically silent in this case. Their significance resides more in the risk they represent as a reservoir for future variants with epidemic potential. Thr and Ala exhibited the highest mutation frequencies, likely due to low structural constraint within the P2 domain. Overall, GII.4 noroviruses evolve in immunodominant epitopes to evade host immunity while preserving key functional regions. These findings support long-term molecular surveillance and rational vaccine design targeting conserved epitopes.

There were several limitations of the study. First, although great effort was taken, only three gene fragments were amplified successfully from nine specimens with Ct values lower than 25 in rRT-PCR. Additionally, there were 15 partial RdRp gene sequences, 14 region C sequences and 11 domain P sequences of VP1 gene from 23 specimens positive for HuNoV. Second, this study included only a single pediatric patient with Burkitt lymphoma, which inevitably limits the generalizability of the conclusions. However, it is precisely this 19-month longitudinal follow-up that provided a rare window into the persistent evolution and quasi-species dynamics of HuNoV in an immunocompromised host. Although the observed pattern of mutation accumulation in the P2 subdomain awaits validation in more cases from diverse clinical contexts, it provides valuable baseline data for understanding viral evolutionary patterns during chronic infection and for monitoring the potential emergence of novel variants. Third, Sanger sequencing has limitations in terms of characterizing intra-host viral clonal diversity, and although mNGS was used for three key samples, it remains insufficient to fully resolve the complex population structure of the virus within the host.

In summary, this study provides detailed data on the intra-host molecular evolution of HuNoV GII.4 Sydney_2012 in a pediatric Burkitt lymphoma patient. Two distinct sequences differing by five amino acids in the P2 subdomain were found in one sample, indicating quasi-species evolution during chronic infection. The intra-host evolutionary rate matched the global population rate, suggesting that prolonged infection drives mutation accumulation. These findings highlight the importance of monitoring HuNoV infection in immunocompromised populations.

## Figures and Tables

**Table 1:** The Ct values of HuNoV nucleic acids determined by rRT-PCR and results of HuNoV gene amplification in the monitoring surveillance.

No.	Type of Specimens	Symptoms at Collection	Date of Collection	Ct Value in rRT-PCR	RdRP	Region C of VP1	P Domain of VP1
N302	Fecal	Vomiting	15 August 2016	15.9	+	+	+
N339	Fecal	Vomiting, Mucous stool	12 September 2016	15.47	+	+	+
N347	Vomitus	Vomiting, Loose stool	13 September 2016	30	−	−	−
N349	Fecal	Fever, Vomiting, Watery diarrhea	15 September 2016	18	+	+	+
N372	Fecal	Fever	25 September 2016	15.74	+	+	+
N468	Fecal	Fever	11 November 2016	15.08	+	+	+
N478	Fecal	Vomiting	21 November 2016	16	+	+	+
N515	Fecal	Vomiting, Watery diarrhea	8 December 2016	17	+	+	+
N556	Fecal	Vomiting	18 December 2016	28	+	+	−
N705	Fecal	Asymptomatic	6 March 2017	19	+	+	+
N743	Fecal	Asymptomatic	15 March 2017	22	+	−	+
N797	Fecal	Asymptomatic	31 March 2017	23	+	+	+
N804	Fecal	Asymptomatic	5 April 2017	27.55	−	−	−
N900	Fecal	Asymptomatic	3 May 2017	25.06	+	+	−
N911	Fecal	Asymptomatic	9 May 2017	27	+	+	−
N982	Fecal	Asymptomatic	5 June 2017	31	−	−	−
N1041-1	Fecal	Asymptomatic	13 July 2017	29.08	+	+	−
N1041-2	Fecal	Asymptomatic	14 July 2017	29	+	+	−
N1053	Fecal	Asymptomatic	17 July 2017	30	−	−	+
N1103	Fecal	Asymptomatic	23 August 2017	32	−	−	−
N1138	Fecal	Asymptomatic	18 September 2017	-	−	−	−
N1188	Fecal	Asymptomatic	16 October 2017	35	−	−	−
N1253	Fecal	Asymptomatic	2 November 2017	-	−	−	−
N1368	Fecal	Asymptomatic	23 November 2017	35	−	−	−
N1407	Fecal	Asymptomatic	30 November 2017	-	−	−	−
N1685	Fecal	Asymptomatic	7 February 2018	-	−	−	−
N1767	Fecal	Asymptomatic	20 March 2018	-	−	−	−

Notes: Ct, cycle threshold. HuNoV, human norovirus. rRT-PCR, real-time reverse-transcription polymerase chain reaction. +, with gene sequence. −, negative for HuNoV or without gene sequence. Symptoms were retrospectively extracted from available clinical records; some time points may have incomplete symptom documentation. “Asymptomatic” indicates no gastrointestinal symptoms were recorded at the time of specimen collection.

**Table 2:** Amino acid mutations with changed polarity in subdomain P2.

Amino Acid Position	Mutation(s)	Polarity Change	Pattern Type	First Appearance (Sample ID, Date)	Last Appearance (Sample ID, Date)
294	Thr → Ala/Arg	+/−	Persistent	N339 (12 September 2016)	N1053 (17 July 2017)
309	Ser → Asn	+/+	Late-appearing persistent	N515 (8 December 2016)	N1053 (17 July 2017)
317	Ile → Thr	−/+	Late-appearing persistent	N515 (8 December 2016)	N1053 (17 July 2017)
333 ^a^	Met → Thr/Val	−/+/−	Late-appearing persistent	N705 (6 March 2017)	N1053 (17 July 2017)
340	Thr → Ala	+/−	Transient	N372 (25 September 2016)	N372 (25 September 2016)
341	Asp → Asn	+/+	Late-appearing persistent	N515 (8 December 2016)	N1053 (17 July 2017)
349	Ala → Thr	−/+	Late-appearing persistent	N515 (8 December 2016)	N1053 (17 July 2017)
365	Ala → Val	−/−	Persistent	N302 (15 August 2016)	N1053 (17 July 2017)
368	Ser → Asn	+/+	Transient	N478 (21 November 2016)	N515 (8 December 2016)
372	Ile → Val	−/−	Transient	N372 (25 September 2016)	N468 (11 November 2016)
377	Ala → Thr	−/+	Persistent	N302 (15 August 2016)	N1053 (17 July 2017)
394	Thr → Ser	+/+	Late-appearing persistent	N515 (8 December 2016)	N1053 (17 July 2017)
447	Met → Ile	−/−	Late-appearing persistent	N705 (6 March 2017)	N1053 (17 July 2017)
460	Tyr → His	+/+	Late-appearing persistent	N705 (6 March 2017)	N1053 (17 July 2017)

Notes: ‘+’ means polar amino acid; ‘−’ means nonpolar amino acid. ‘^a^’ means alternating between Thr and Val.

## Data Availability

The original contributions presented in this study are included in the article/Supplementary Material. Further inquiries can be directed to the corresponding authors.

## Supplementary Materials

The following supporting information can be downloaded at: https://www.mdpi.com/article/10.3390/v18050538/s1, Figure S1. Sanger sequencing chromatograms of the P2 subdomain from sample N705. Arrows indicate double peaks at specific nucleotide positions. Red boxes denote amino acid codons within this region. The first G within the red box corresponds to nucleotide position 1171 of the VP1 gene.
