# Supplementary material for: A Prolonged Norovirus Infection and the Molecular Evolution of Human Norovirus Within-Host in a Child with Burkitt Lymphoma
Source: Viruses. 2026 May 7;18(5):538. doi: 10.3390/v18050538 (PMC13211706; doi:10.3390/v18050538)
Supplement: Supplementary file 1 [file viruses-18-00538-s001.zip › viruses-4298837-supplementary.pdf]

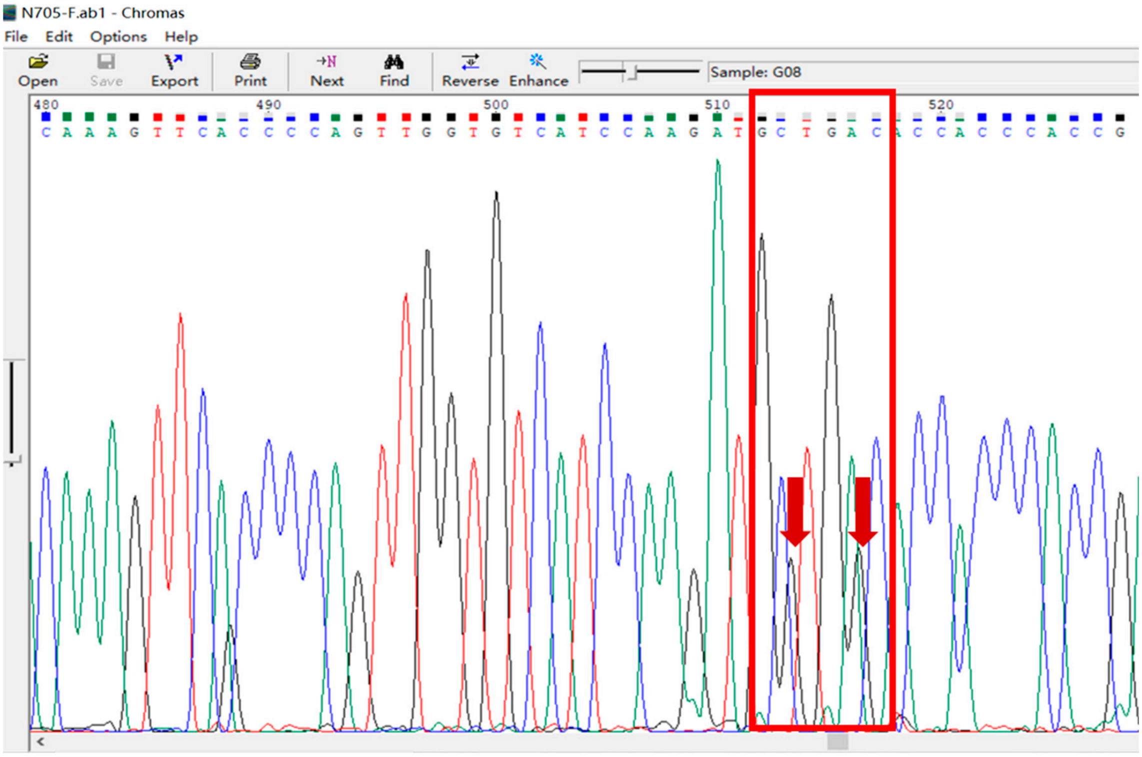

Supplementary Figure S1. Sanger sequencing chromatograms of the P2 subdomain from sample N705.

Arrows indicate double peaks at specific nucleotide positions. Red boxes denote amino acid codons within this region. The first G within the red box corresponds to nucleotide position 1171 of the VP1 gene.
